# Ellagitannins in Cancer Chemoprevention and Therapy

**DOI:** 10.3390/toxins8050151

**Published:** 2016-05-13

**Authors:** Tariq Ismail, Cinzia Calcabrini, Anna Rita Diaz, Carmela Fimognari, Eleonora Turrini, Elena Catanzaro, Saeed Akhtar, Piero Sestili

**Affiliations:** 1 Institute of Food Science & Nutrition, Faculty of Agricultural Sciences and Technology, Bahauddin Zakariya University, Bosan Road, Multan 60800, Punjab, Pakistan; ammarbintariq@yahoo.com (T.I.); saeedbzu@yahoo.com (S.A.); 2 Department of Biomolecular Sciences, University of Urbino Carlo Bo, Via I Maggetti 26, 61029 Urbino (PU), Italy; anna.diaz@uniurb.it; 3 Department for Life Quality Studies, Alma Mater Studiorum-University of Bologna, Corso d'Augusto 237, 47921 Rimini (RN), Italy; cinzia.calcabrini@unibo.it (C.C.); carmela.fimognari@unibo.it (C.F.); eleonora.turrini@unibo.it (E.T.); elena.catanzaro2@unibo.it (E.C.)

**Keywords:** ellagitannins, phytochemicals, cancer, chemoprevention, cancer therapy, safety

## Abstract

It is universally accepted that diets rich in fruit and vegetables lead to reduction in the risk of common forms of cancer and are useful in cancer prevention. Indeed edible vegetables and fruits contain a wide variety of phytochemicals with proven antioxidant, anti-carcinogenic, and chemopreventive activity; moreover, some of these phytochemicals also display direct antiproliferative activity towards tumor cells, with the additional advantage of high tolerability and low toxicity. The most important dietary phytochemicals are isothiocyanates, ellagitannins (ET), polyphenols, indoles, flavonoids, retinoids, tocopherols. Among this very wide panel of compounds, ET represent an important class of phytochemicals which are being increasingly investigated for their chemopreventive and anticancer activities. This article reviews the chemistry, the dietary sources, the pharmacokinetics, the evidence on chemopreventive efficacy and the anticancer activity of ET with regard to the most sensitive tumors, as well as the mechanisms underlying their clinically-valuable properties.

## 1. Introduction

Despite the enormous efforts of the scientific and medical community, cancer still represents the second leading cause of death and is nearly becoming the leading one in the elderly [1]. It is estimated that, due to demographic changes alone, in the next 15 years the number of new cancer cases will increase by 70% worldwide [2].

The lack of effective diagnostic tools for early detection of several tumors, the limited treatment options for patients with advanced stages of cancer, and the onset of multiple drug resistance favor poor prognosis and high mortality rates. The significant, but still unsatisfactory, improvement of survival, the severe toxicity profile, and the high costs characterizing many current anticancer therapies clearly show that a threshold in terms of clinical benefit and patients’ tolerance has been reached. Thus, the identification and development of innovative, preventive as well as therapeutic strategies to contrast cancer-associated morbidity and mortality are urgently needed.

**Figure 1:**
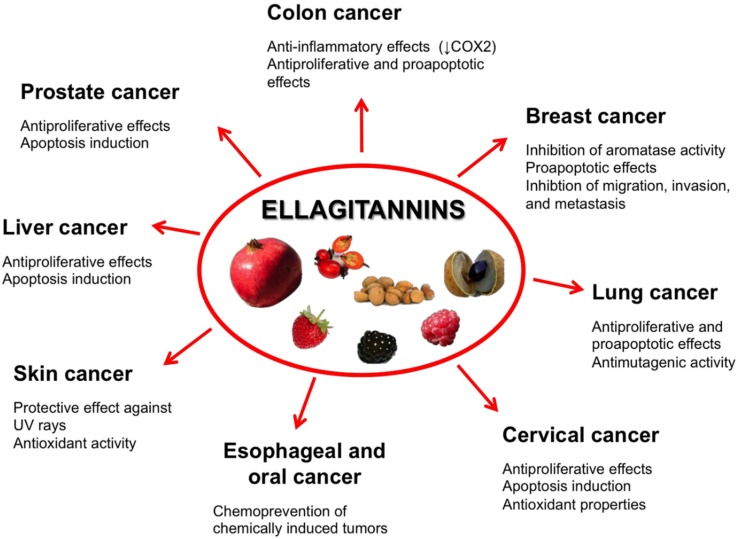
Fruits containing ET with chemopreventive, anticarcinogenic, and antiproliferative activities.

Epidemiological, preclinical, and clinical studies have generally concluded that a diet rich in phytochemicals can reduce the risk of cancer [2,3]. Due to their pleiotropism which includes antioxidant, anti-inflammatory, and antiproliferative activities as well as modulatory effects on subcellular signaling pathways, phytochemicals from edible fruits and vegetables are recognized as an effective option to counteract cancer incidence and mortality [3,4,5]. Plants constitute a primary and large source of various chemical compounds including alkaloids, flavonoids, phenolics, tannins, tocopherols, triterpenes, and isothiocyanates. Ellagitannins (ET) are an important class of phytochemicals contained in a number of edible plants and fruits recommended by the traditional medicine of a variety of cultures, both in the developing and developed countries, to treat common health problems. ET biological and nutraceutical potential has received increasing attention over the last several decades. ET exert multiple and clinically-valuable activities [4], and among them the chemopreventive, anticarcinogenic, and antiproliferative activities are being receiving growing interest and attention (Figure 1).

## 2. Dietary Sources, Types, and Occurrence

**Figure 2:**
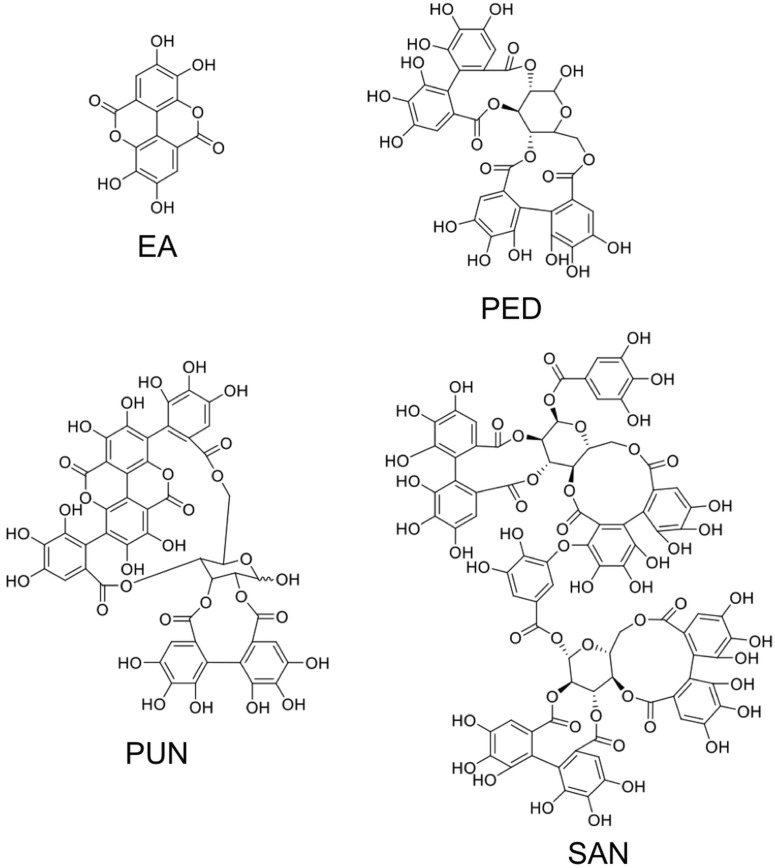
Structures of ellagic acid and of three major and representative ellagitannins. EA, ellagic acid; PED, pedunculagin; PUN, punicalagin; SAN, sanguiin H-6.

ET and their derivatives are noticeably contained in edible seeds, nuts, and various fruits of nutritional interests. The structures of relevant ETs and of ellagic acid are shown in Figure 2. A wide variety of fresh fruits including berries, like raspberries, black raspberries, strawberries, pomegranate, longan, and dried nuts, are renowned for their ample polyphenols concentration in the form of ET [5]. Five species of berries including raspberry, strawberry, cloudberry, rose hip, and sea buckthorn were identified by Koponen *et al.*, [6] as significant carrier of ET in a range of 1–330 mg per 100 g of fruit. Sanguiin H-6 and lambertianin C were reported from Glen Ample raspberries and Scottish-grown red raspberries, along with some trace levels of ellagic acid [7,8]. Blackberries (fruit and seeds) have been reported for a range of ET including pedunculagin, casuarictin, sanguiin H-6 (lambertianin A), and lambertianin (C and D) [9,10,11]. Pomegranate and various fractions of the fruit are known for their cancer chemopreventive properties owing to their unique phenolics composition in the form of ET, which include punicalagin, punicalin, granatin A, granatin B, tellimagrandin I, pedunculagin, corilagin, gallagic acid, ellagic acid, and casuarinin [12].

ET, predominately those isolated from pomegranate (e.g., punicalagin), have gained a wide popularity as preventive and therapeutic ethnopharmacological approaches for cancer treatment. However, a lot more has been added to this class of compounds from fruits other than pomegranate, including raspberries, blueberries, strawberries, muscadine grapes, and longan [7,13,14,15,16,17,18]. Major phenolic fractions recovered from longan include gallic acid, ellagic acid, and corilagin, much more concentrated in the seed segment as compared to the fruit pulp and peel [17]. Good essential fatty acid composition of nuts and fairly high concentrations of ET and their derived fractions, such as ellagic acid and its glycosidic derivatives have been associated with the potential cardioprotective properties of nuts. Ellagic acid (free and total) has been reported in a range of 0.37–823 mg per 100 g of dried nuts [19]. High concentrations of a variety of ET (ellagic acid, sanguiin H2 and 6, lambertianin C, castalagin/vescalagin, galloyl-bis-HHDP glucose, pedunculagin) can be found in blackberries (*Rubus* sp.) [20]. Shi *et al.*, [21] identified agrimoniin as the second highest phenolic compound of strawberries.

Irrespective of the edible fractions of fruiting plants, some inedible fractions like fruit peels, bark and foliage have also been reported as good source of hydrolysable tannins including bioactive ET [4,22]. Leaves extracts of *Shepherdia argentea*—a deciduous shrub commonly known as silver buffaloberry—were reported as a good reserve of gluconic acid core carrying the potential anti-HIV novel ET, such as hippophaenin A, shephagenin A and shephagenin B [23].

## 3. Ellagitannins—Classification and Chemistry

Tannins are unique secondary metabolites of plant phenolics with relatively higher molecular weight (300–30,000 Da) and bear the ability to generate complexes with some macromolecules, like proteins and carbohydrates [24]. Chemistry and nomenclature of the tannins is complicated by virtue of the frequent changes which parallel the advancement in this very specific field [25]. Taking into account different definitions of tannins [26,27], these compounds may be referred as either galloyl esters and their derivatives (ET, gallotannins, and complex tannins), or the oligomeric and polymeric proanthocyanidins (condensed tannins). In a broader perspective, tannins may be classified most satisfactorily and unambiguously on the basis of structural configuration and/or solubility [28]. C–C coupling of galloyl units in absence of glycosidically-linked catechin make ETs structurally different from the condensed tannins that are characterized by monomeric catechin linkages (C4–C8 or C4–C6) to generate oligomeric likewise polymeric proanthocyanidins [27]. Gallotannins and ETs constitute a major group of tannins *i.e.*, hydrolysable tannins that are well known for their properties to hydrolyze into hexahydroxydiphenol (HHDP) or gallic acid moieties. Gallotannins are the gallic acid derivatives carrying ≥ six gallyol groups and might further be characterized on account of one or more than one digalloyl group [29].

ETs (hydrolysable tannins) on their hydrolysis yield gallic acid and ellagic acid from the compounds carrying gallyol groups and HHDP groups, respectively [28]. *In vitro* digestion models declare ETs to remain stable under the normal physiological condition of the stomach [30]. However, ETs hydrolysis to free ellagic acid or their degradation may proceed in the small intestine at neutral to alkaline pH [31]. Biologically, condensed tannins and gallotannins are thought to deliver relatively higher protein precipitation properties as compare to the ETs and hence are considered potential antinutritional compounds from the class of plants polyphenolics [32]. Gallotannins and condensed tannins have also been reported as oxidatively least active tannins as compared to the ETs and on the same time gallotannins and condensed tannins have also been found to reduce pro-oxidant properties of ETs [33,34].

### 3.1. Simple Ellagitannins

ET (M.W. 300–20,000 Da) are non-nitrogenous compounds with at least two C–C coupled galloyl units with no glycosidically-bonded catechin unit [3,35]. ET are derivatives of 1,2,3,4,6-penta-*O*-galloyl-β-d-glucopyranose (PGG). Structurally, ET are esters of carbohydrates and or cyclitols and also include metabolic compounds derived from oxidative cleavage of either condensed or hydrolysable tannins [27,35,36]. The presence of hexahydroxydiphenol (HHDP) in a glucopyranose ring in addition to acyl units and certain HHDP metabolites such as dehydrohexahydroxydiphenol (DHHDP), valoneoyl and chebuloyl groups constitute simple ET. Tellimagrandin I and II, pedunulagin, casuarictin, and chebulagic acid originate from the specific orientation and number of acyl groups on glucose units. Variation in HHDP group originates by linking (C–C or C–O) one or more galloyl groups to HHDP unit.

Structural diversity of ET has been reported to correlate with their carrier-plants’ taxonomy and evolutionary hierarchy [37]. More often, monomeric ET or oligomeric ET constitute the major tannic component of plant species. The monomeric compounds of the group include tellimagrandins I and II, pedunculagin, casuarictin, and potentillin. Type I hydrolysable tannins (*i.e.*, gallotannins) carrying HHDP in stable conformation at either the 2,3 or 4,6 position on a d-glucopyranose may be referred to as a simple ET [38,39,40]. Geraniin, a type III ET, is another example of monomeric simple ET carrying a DHHDP unit linked to d-gluopyranose of HHDP unit via ^1^C_4_ conformation. Dimers of ET are generated by intermolecular oxidative coupling/condensation of simple ET.

### 3.2. Glycosidic Ellagitannins

Chemically, the *C*-glycosidic linkage of ET is established via intermolecular bonds between two monomeric units, one carrying anomeric carbon while the second one galloyl or HHDP group [3,35,41]. Most recently *C*-glycosidic ET including granadinin, vescalagin, methylvescalagin, castalagin, stachyurin, and casuarinin have been reported from the peel and seed fraction of camu-camu, a fruiting tree of Amazon rainforest [42]. Woody fractions of various fruits, particularly the nuts and berries, have also been observed to hold novel *C*-glycosidic ET (e.g., castalagin and vescalagin). Castacrenins D and F are two other forms of *C*-glycosidic ET isolated from the woody fraction of Japanese chestnut and carry gallic acid/ellagic acid moieties [43]. Treating vescalagin with *Lentinula edodes* generates quercusnins A and B that may be referred as fungal metabolites of *C*-glycosidic ET [44]. Castacrenins D and F isolated from chestnut wood may generate oxidative metabolites, namely castacrenins E and G, by replacing pyrogallol rings of *C*-glycosidic ET with cyclopentone rings [43]. Rhoipteleanins H, I, and J were reported as novel *C*-glycosidic ET isolated from the fruit and bark fractions of *Rhoiptelea chiliantha.* Structural configuration of rhoipteleanins H revealed the presence of cyclopentenone carboxy moieties that are generated by oxidation and rearrangement of *C*-glycosidic ET aromatic ring [45].

Condensate of *C*-glycosidic ET is another subclass of hydrolysable tannins, which includes rhoipteleanin J produced by the intermolecular condensation (C–C or C–O) of monomeric *C*-glycosidic ET followed by oxidation of aromatic rings of ET [45]. Wine aged in oak wood barrels is often reported to carry oak ET, particularly the condensation products of monomeric *C*-glycosidic ET. The studies infer *C*-glycosidic ET to play a significant role in modulation of organoleptic features of wine aged in oak wood barrels [46].

## 4. Ellagitannins Pharmacokinetics

A precise knowledge of phytochemicals’ pharmacokinetics is very important to exploit their health benefits, as well as the effects of their metabolites [47]. *In vivo*, ET, instead of being absorbed directly into the blood stream, are physiologically hydrolyzed to ellagic acid, which is further metabolized to biologically-active and bioavailable derivatives, *i.e.*, urolithins, by the activity of microbiota in gastrointestinal (GI) tract [5,48]. The biological properties of ET, such as free radical scavenging, further depend on their metabolic transformation inside gut. ET recovered from pomegranate juice may be metabolically converted by gut microbiota to urolithin A, B, C, D, 8-*O*-methylurolithin A, 8,9-di-*O*-methylurolithin C, and 8,9-di-*O*-methylurolithin D, and some of these metabolites display higher antioxidant activity than the parental tannins themselves. For instance, urolithin C and D show an antioxidant capacity—as determined in a cell-based assay—which is 10- to 50-fold higher as compared to punicalagin, punicalin, ellagic acid, and gallic acid [49]. This finding suggests that intestinal transformation products of ET are likely to play a central role for the antioxidant properties at least inside the GI tract. Significant differences in urolithins’ profiles in individual human subjects feed on raspberries—a renowned source of ET—have been attributed to gut microflora, whose variations on an inter-individual basis affect their capacity of hydrolyzing ET and subsequent metabolite synthesis [48,50]. The interaction of gut microbiota composition and the host endogenous excretory system is also likely to play a further role in the observed inter-individual variability [51]. ET are highly stable under the acidic environment of stomach, and retain their composition without being hydrolyzed to simpler compounds when exposed to various gastric enzymes. Consequently the complex structure of ET impedes their gastric absorption: however, the stomach might serve as the first site of absorption of free ellagic acid and pre-hydrolyzed forms of ET.

Contrary to stomach, the neutral or alkaline environments of duodenum and small intestines, characterized by pH values ranging from 7.1 to 8.4, allow ET hydrolyzation [31,41]. In humans, ET are rapidly absorbed and metabolized, as documented by [18,52]: following ingestion of pomegranate juice (at a dose containing 25 mg of ellagic acid and 318 mg of ET), ellagic acid can be found in plasma for up to 4 h while, at later times, it is no more detectable. In contrast, another study reported that no ellagic acid could be detected in plasma during the 4 h following the juice intake [53], a discrepancy which has been attributed to inter-individual variability [54]. Ellagic acid is converted by catechol-*O*-methyl transferase to dimethylellagic acid, which is then glucuronidated and excreted [52].

Finally, the microbiologically metabolized fraction of ET, *i.e.*, urolithins, is further incorporated to enterohepatic circulation system [18,53,55,56].

## 5. Ellagitannins for Tumor Chemoprevention and Therapy

The development of novel mechanism-based chemopreventive and antitumor approaches to fight cancer through the use of dietary substances which humans can easily accept has become an important goal. Along this line, ET have received increasing attention over the last two decades.

Similarly to other anticancer phytochemicals, ET display chemopreventive and chemotherapeutic activities [3]. The chemopreventive activity of ET and derivatives, such as ellagic acid has been primarily associated with their antioxidant capacity, that varies with the degree of hydroxylation [5,57] and depends from both a direct radical scavenging and iron chelation activity.

The well-known anti-inflammatory capacity represents another important feature of ET chemopreventive and antitumor activity [55], that being persisting inflammation involved both as a causative and a facilitating factor in carcinogenesis and cancer development [58]. For example, pomegranate ET inhibit pro-inflammatory pathways including, but not limited to, the NF-κB pathway, whose activation leads to immune reactions, inflammation, and the transcription of genes involved in cell survival, such as Bclx and inhibitors of apoptosis. Constitutive activation of NF-κB has been observed in prostate cancers, where it sustains chronic inflammation and promotes the development of high-grade prostate cancer. With respect to inflammation, it is worth noting that, similarly to many polyphenols, the antioxidant activity of ET participates to an “anti-inflammatory loop” with other mechanisms, since it lowers the levels of radicals which otherwise would act as pro-inflammatory stimuli.

The direct antiproliferative effects of ET have been attributed to multiple mechanisms (see the next subchapters) including the cell cycle arrest capacity and the properties enabling cancer cells to follow apoptosis through the mitochondrial route and self-destruction after replication [59,60,61,62]. In addition to directly targeting tumor cell survival, the cytotoxic/cytostatic activities of ET might also concur with the chemopreventive potential, since they prevent tumor cells from converting into more malignant phenotypes and from replicating.

A study on 1,3-di-*O*-galloyl-4,6-(s)-HHDP-β-d-glucopyranose (an ET from *Balanophora japonica* MAKINO) points to the complexity and multiplicity of the mechanisms contributing to the anticancer activity of ET, *i.e.*, the same complexity and multiplicity characterizing also other classes of phytochemicals. Indeed, the antiproliferative activity of 1,3-di-*O*-galloyl-4,6-(s)-HHDP-β-d-glucopyranose in human Hep-G2 liver cancer cells was also associated to an altered regulation of 25 miRNAs including the let-7 family members miR-370, miR-373, and miR-526b, identified as likely targets with roles in cell proliferation and differentiation [63]. The fact that in cell culture systems combinations of ET or of ET and other phytochemicals present in plant or fruit extracts are more cytotoxic than any single ET [64], is suggestive of the multifactorial effects, chemical synergy, and multiplicity of the mechanisms behind their antitumor activity. To this regard, the capacity of some ET to inhibit angiogenesis, a fundamental event accompanying tumor growth, both in *in vitro* and *in vivo* prostate cancer models [65], and to reduce endothelial cell growth through binding to vascular endothelial growth factor receptors [66] represents a further and significant antitumor mechanism.

In analogy to other polyphenols, ET could also be utilized to increase the sensitivity of tumor cells to standard chemotherapeutic drugs [67], with the aim of obtaining an increase of their antitumor efficacy along with a reduction of their doses and, consequently, of their severe adverse effects which often represent a limiting factor for the prosecution of the therapeutic regimens.

As a premise to the literature data discussed in the next paragraphs, it is important noting that, since ET are not absorbed systemically after oral administration as such [48], the studies where ET extracts were given to cultured cancer cells are unlikely to be predictive of the effects which could be attained after oral ingestion *in vivo*. Rather these data could be representative of intravenously administered ET, but the toxicology of this administration route is not known.

The next sections of the review will discuss more in depth the ET anticancer mechanisms and properties emerging from *in vivo* and *in vitro* studies on a panel of tumors or tumor cells which appear as potentially sensitive targets for these phytochemicals.

### 5.1. Prostate Cancer

Prostate cancer is the second leading cancer-associated death risk factor among U.S. males [68]. Phytochemicals originating from various food sources slow down the progression of prostate cancer, whereas a majority of other nutrients are reported to be non-effective in either preventing or curing prostate cancer [69]. Evidence-based findings support the consolidated role of fruits, vegetables, and various culinary herbs of different cultures in averting various forms of cancers, but relatively weak and inconsistent relationships have been presented so far for prostate cancer [70,71]. Somehow more promising seem to be the edible fruits containing high amounts of ET, which have been extensively tested *in vivo* for their prostate cancer inhibitory properties. As it has been shown in animal models, higher concentrations of ET are recorded in prostate and colon tissues as compared to the others [72]. Pomegranate holds one of the highest concentration of ET [55]. Antitumor activities of pomegranate fruit juice, peel extracts, and seed oil have been reported against prostate cancer cells [73]. Dose-dependent anti-proliferative and pro-apoptotic effects of pomegranate fruit extracts (10–100 µg/mL) have been documented against aggressive human prostate cancer cells (PC3) [74]: induction of pro-apoptotic mediators (Bax and Bak), downregulation of Bcl-2 and Bcl-XL, and reduced expression of cyclin-dependent kinases 2, 4, 6, and cyclins D1, D2, and E have been identified as the mechanisms responsible for these effects.

Pomegranate extract inhibited proliferation of endothelial (HUVEC) and prostate (LNCaP) cancer cells; the extract also reduced LNCaP prostate cancer xenograft size, tumor vessel density, VEGF peptide levels and HIF-α expression after four weeks of treatment in severe combined immunodeficient mice [65].

Oenothein B, a macrocyclic ET, and quercetin-3-*O*-glucuronide from *Epilobium* sp. herbs—used in traditional medicine to treat benign prostatic hyperplasia and prostatic adenoma—have been proven to strongly inhibit the proliferation of human prostate cancer cells [75]. *Hibiscus sabdariffa* leaf extracts, which contain high amounts of ellagic acid, have been reported to inhibit the growth [76] and the expressions of metastasis-related molecular proteins [75] of LNCaP cells via activation of the mitochondrial pathway and suppression of the Akt/NF-kB signaling pathway, respectively. *Terminalia chebula*—a common ayurvedic ethnic drug of the Indian subcontinent—has been recognized for its potential biological and pharmacological properties [77]. Chebulinic acid is the predominant and more characteristic ET among the various constituents of chebula fruit (*T. chebula*). Methanolic extract (70%) of *T. chebula* fruits was shown to inhibit proliferation and induce cell death in PC3 prostate cancer cells as well as in PNT1A non-tumorigenic human prostate cells in a dose-dependent manner [78]. At low concentrations, the extract promoted initiation of apoptotic cell death, while at higher doses necrosis was the predominant type of cell demise; chebulinic acid, tannic acid, and ellagic acid were the most cytotoxic phenolics, and are likely responsible for the antitumor activity of *T. chebula* fruit extracts [78].

### 5.2. Colon Cancer

Cancer statistics, as reported from Centers for Disease Control and Prevention, rate colon cancer as the fourth highest death factor in USA [68]. It is widely accepted that herbal sources may provide therapeutically relevant compounds for the management of colorectal cancers. In this regard, it is worth noting that World Health Organization estimates over 80% of the entire world population rely on biomolecules with broad ethnopharmacological properties as a primary health care solution [79].

The strict correlation between chronic inflammation, malignant transformation and development of colorectal cancer is widely recognized [80]. Indeed, non-steroidal anti-inflammatory drugs have proven to be effective in preventing the formation of colorectal tumors and their malignant transformation in both preclinical and clinical studies [81]. However, unwanted, sometimes severe or even fatal, side effects (ulceration, renal toxicity, gastric bleeding) represent a major limitation for the use of these synthetic drugs: in search of alternative therapeutic options, exploration and utilization of natural biomolecules as anti-inflammatory formulations are in progress [82]. Various phytochemicals modulate inflammatory cell signaling in colon cancer: among them, pomegranate ET (*i.e.*, punicalagin and ellagic acid) have been shown to suppress cyclooxygenase-2 (COX-2) protein expression in human colon cancer (HT-29) cells [83]. Exposing HT-29 cells to 50 mg/L of powdered pomegranate juice, total pomegranate ET, or punicalagin reduces the expression of COX-2 protein by 79%, 55%, and 48%, respectively, and inhibits production of pro-inflammatory prostaglandins [83]. Another study conducted by Kasimsetty *et al.*, [84] reported that pomegranate ET and their metabolites, *i.e.*, urolithins A and C, inhibit HT-29 cells proliferation via G0/G1 and G2/M arrest, followed by induction of apoptosis. Interestingly, urolithins display advantageous pharmacokinetics over other agents, in that they tend to persist in the colon through enterohepatic circulation. Scarce information is available on the mechanistic role of ET and their metabolites, mainly urolithins, in colon cancer chemoprevention. Sharma *et al.*, [85] showed that fruit ET and their metabolites inhibit canonical Wnt signaling pathway, which is involved in the development of the majority (~90%) of colon cancers. In this light, ET and their colon-derived metabolites may be most relevant in relation to cancer prevention rather than treatment. To this regard, it is important noting that the concentrations of ET and their metabolites such as ellagic acid or urolithin A resulting in a 50% inhibition of Wnt signaling in 293T human colon cancer cells are comparable with those nutritionally attainable after regular consumption of ET-rich fruits or beverages [85].

### 5.3. Breast Cancer

Breast cancer is the most prevalent, spontaneous hormone-associated malignancy in women and is the most common gender-related cause of death around the globe [86,87]. Estrogen is the major stimulating factor of breast cancer cells’ proliferation and tumor cells’ growth. Upregulation of growth hormone receptors in breast malignant cells, as compared to the normal breast tissue, points to the key role of the pituitary, as well as the growth hormones, in the development of breast cancer in humans [88].

Complementary and alternative medicines in the form of bioactive fractions and raw decoctions of herbs, edible and inedible segments of various fruits and vegetables, are under assessment for their potential in treating breast cancer [89]. Pomegranate, its juice, and other fractions of the fruit are the richest source of high-molecular-weight ET, in particular punicalagin, as compared to any other known and commonly-consumed fruit [55]. Estrogen-induced expression of peptides growth factors is the major concern in the development and growth of estrogen-responsive mammary cancer: inhibition of this circuitry is the rationale for the use of antiestrogens and aromatase inhibitors to treat these types of breast cancer [90,91]. Pomegranate ET-derived compounds have been shown to block endogenous estrogen synthesis by inhibiting aromatase activity. Polyphenol-rich fractions derived from fermented juice, aqueous pericarp extract and cold-pressed or supercritical CO_2_-extracted seed oil of pomegranate (Wonderful cultivar) have been reported to inhibit aromatase and 17-beta-hydroxysteroid dehydrogenase type 1 (a key determinant of the increase in estradiol/estrone ratio) activities [92]. The same authors found that the polyphenol-rich fractions from fermented juice and pericarp inhibited the viability of MCF-7 estrogen-dependent tumor cells to a higher extent as compared to estrogen-independent MB-MDA-231 cells; interestingly, normal human breast epithelial cells (MCF-10A) were far less sensitive to the inhibitory effect of polyphenol-rich fractions. Among some other fruits, the ripened fruit and seeds of *Syzygium cumini* (commonly known as *jamun* in Indian subcontinent culture) and *Eugenia jambolana* have also been reported as good reservoir of ellagic acid/ET which, in addition to anthocyanins, can exhibit anti-proliferative properties against various cancer cells [93]. Accordingly, and in strict analogy with the study by Kim *et al.*, [92], *Jamun* fruit extracts have been shown to inhibit over-expressing aromatase and estrogen-dependent MCF-7aro cell proliferation (IC50 27 µg/mL) more effectively as compared to estrogen receptor-negative MDA-MB-231 (IC50 40 µg/mL) breast cancer cells [94]. Pro-apoptotic effects were observed (200 µg/mL) against both MCF-7aro and MDA-MB-231 breast cancer cells, but not toward the normal MCF-10A breast cells.

Upregulation of the phosphoinositide-3 kinase (PI3K)/Akt signaling pathway is a common feature in most human cancers, including breast cancer. Targeting the PI3K pathway with small molecule inhibitors has been studied for therapeutic purposes, and inhibitors such as GDC-0941 or GDC-0980 have entered preclinical trials [95].

*Cistaceae* family—rock rose family—has been traditionally used in Mediterranean cultures since ancient times. Aqueous extracts recovered from the leaves of *C. populifolius*, which contain high amounts of punicalagin and other ET, have been shown to be cytotoxic against HER 2-dependent (MCF 7/HER2) and -independent (JIMT-1) human breast cancer cells [96]. Since JIMT-1 cells are representative of trastuzumab-resistant cells, *C. populifolius* extracts may be important in the treatment of breast tumors insensitive to this targeted drug.

Finally, oenothein B has proven to exert *in vitro* inhibitory properties against mammary ascites tumors (MM2) cells and Meth-2 solid tumors by releasing interlukin-1 and interlukin-1β-like cytokines [97].

### 5.4. Oral, Esophageal, and Gastric Cancers

Enzinger and Mayer [98] in their report published in the New England Journal of Medicine indicated esophageal cancer as the deadliest and least-studied type of cancer, with relatively small advancements in diagnosis and treatment over a three decades period. Among other etiological factors of esophageal cancer, inhalation of cigarette smoke is the most obnoxious one in exposing esophageal mucosa to potential carcinogens (*i.e.*, nitrosamines) [99]. Fruits, particularly berries, are a good source of antioxidant including vitamins, anthocyanins, ET, and a wide range of phenolic acids [100]. Consumption of fruits and vegetables has been linked with lower risks of gastrointestinal tract cancer development. This is one of the reasons that prompted researchers to exploit the nutraceutical potential of berries and their biomolecules as chemopreventive food and dietary supplements [101].

As demonstrated by Yoshida *et al.*, [23], high molecular weight oligomeric ET (eucarpanins and elaeagnatins) and macrocyclic dimers including camelliin B, oentothein B, and woodfordin C have cytotoxic properties and induce apoptosis through a pro-oxidant mechanism in tumor cells of oral squamous cell carcinoma (HSC-2, HSG) to a higher extent as compared to normal fibroblasts. These ET are contained in high amounts in flowering plants of *Myrtaceae* and *Elaeagnaceae* family. Black raspberries possess conspicuous quantities of anthocyanins and ET that make them rational candidates for a preventive and therapeutic approach against certain GI tract cancers [102]. Previous studies by Mandal and Stoner [103] and Daniel and Stoner [104] demonstrated that ellagic acid (4 g/kg b.w.) significantly decreased (~60%) the number of *N*-nitrosomethylbenzylamine (NBMA)-induced esophageal tumors in rats. Latter work by Stoner and Morse [105] confirmed the potent anti-tumorigenic property of ellagic acid in rats exposed to NMBA and tobacco nitrosamines through the inhibition of cytochrome P450, which is responsible for the metabolic activation of these carcinogens. Another study by Stoner *et al.*, [100] showed that a lyophilized mix of berries (black raspberries, blackberries, and strawberries) inhibits tumor initiation and progression via downregulation of COX-2 and inducible nitric oxide synthase, events leading to reduced prostaglandin production and nitrate/nitrite levels in the esophagus, respectively.

In a more recent study [106], NBMA-treated rats fed 5%–10% freeze-dried black raspberries showed fewer hyperplastic and dysplastic esophageal lesions, reduced tumor incidence (~54%), multiplicity (~62%), and proliferation as compared to NBMA control rats; more interestingly, it was shown that black raspberries modulate the expression of a panel of genes and proteins involved in the late stages of NMBA-induced rat esophageal tumorigenesis, such as genes involved in carbohydrate and lipid metabolism, cell proliferation and death, inflammation, and proteins involved in cell-cell adhesion, cell proliferation, apoptosis, inflammation, angiogenesis, and both COX and lipoxygenase pathways of arachidonic acid metabolism.

However, the question of which is the relative contribution of ET and anthocyanins to the above chemopreventive activity of berries in esophageal cancer is still open. Indeed, a study by Wang *et al.*, [107] reported that different berries suppress NMBA-induced tumorigenesis irrespective of their ET and anthocyanin content. This finding suggests that also other components of the active preparations of berries, such as lignans and fibers, contribute to the whole chemopreventive capacity, which does not necessarily coincide with the simple sum of the intrinsic activity of each active constituent, but rather depends on positive (or negative) interactions occurring at specific proportions.

Gemin A and B, two ET from *Geum japonicum* Thunb., were found to exert mild cytotoxic effects on human BGC-823 gastric cancer cells [108].

As to oral cancer, Zhang *et al.*, reported that strawberry crude extracts or their isolated components including ellagic acid were toxic toward human oral CAL-27 and KB tumor cells [109]; ellagic acid alone (50–200 µM) exhibited selective cytotoxicity against HSC-2 oral carcinoma cells [110].

Lyophilized strawberries (LS), which carry 42.9% ET and their derivatives and 48.8% anthocyanins, have been referred as an effective option to prevent oral carcinogenesis: indeed a diet containing 5% LS reduced the number of 7,12-dimethylbenz[a]anthracene (DMBA)-induced cheek pouch tumors in hamsters inhibiting Ras/Raf/ERK-dependent cell proliferation, VEGF-dependent angiogenesis, 5-LOX/LTB4 pathway, and prevented oxidative damage [111]; LS was also found to modulate the genetic signature related to DMBA-induced tumor development, such as p13^Arf^, p16, p53, and Bcl-2 [112].

In the same experimental model of hamster buccal pouch carcinoma, it was demonstrated that dietary supplementation of ellagic acid (up to 0.4%) modulated the expression profiles of 37 genes involved in DMBA-induced oral carcinogenesis [113], blocked the development of carcinomas by suppression of Wnt/β-catenin signaling associated with the inactivation of NF-κB and modulation of key components of the mitochondrial apoptotic network [114], and prevented angiogenesis by abrogating hypoxia-driven PI3K/Akt/mTOR, MAPK, and VEGF/VEGFR2 signaling pathways. These effects were mediated by the suppression of histone deacetylase 6 and HIF-1α responses [115].

By virtue of these properties, LS and its major component ellagic acid are considered among the most important and attractive nutraceutical tools for the prevention of oral cancer [116].

### 5.5. Liver Cancer

Primary liver cancer is, globally, the sixth most frequent cancer, and the second leading cause of cancer death, with a 17% five year survival rate; the leading cause of liver cancer is cirrhosis due to either hepatitis B, hepatitis C, or alcohol [117].

PGG, a major component of *Paeonia suffruticosa* ANDREWS and from *Rhus chinensis* Mill, was found to exhibit *in vitro* antiproliferative activity on human SK-HEP-1 hepatocellular carcinoma cells [118]. The growth-inhibitory effect was related to the ability to cause a G0/G1-phase arrest and to suppress the activation of NF-κB, likely via an IκB-mediated mechanism. PGG was also shown to induce atypical senescence-like S-phase arrest in HepG2 and Huh-7 human hepatocarcinoma cells at sub-lethal doses, increased senescence-associated β-galactosidase activity, and loss of proliferative capacity, through a mechanism involving intracellular generation of oxygen free radicals [119]. No evidence of necrosis or apoptosis was noticed in this study. Interestingly, a more recent report from the same group showed that autophagy was involved in the PGG-induced senescence-like growth arrest, and that activation of MAPK8/9/10 (mitogen-activated protein kinase 8/9/10/c-Jun *N*-terminal kinases) was an essential upstream signal for autophagy to occur [120]; interestingly, these *in vitro* results were also validated *in vivo* in a xenograft mouse model of human HepG2 liver cancer.

Intraperitoneal administration of corilagin from *Phyllanthus urinaria* was found to significantly reduce the *in vivo* growth of xenografted Hep3B hepatocellular carcinoma cells in athymic nude mice with no adverse effects on liver [121]. Corilagin inhibited the growth of normal or tumor hepatic cells with remarkably different IC50s: indeed the values for normal Chang-liver cells *vs.* the hepatocarcinoma cell lines Bel7402 and SMMC7721 were 131.4 *vs.* 24.5 and 23.4 µM, respectively [122]. The antiproliferative effect in SMMC7721 cells was causally associated with arrest at the G2/M phase by the activation of phospho-p53-p21^(Cip1)^-cdc2/cyclin. Furthermore, a 47.3% growth inhibition was recorded in hepatocarcinoma MHCC97-H cells xenografted in Balb/c mice intraperitoneally treated with 30 mg/kg b.w. corilagin for five weeks.

In a parallel, but different direction, corilagin was found to enhance the cytotoxicity of the reference antitumor drugs cisplatin and doxorubicin on Hep3B hepatoma cells at nutritionally-attainable concentrations [67]. The association of corilagin with low dosages of standard anticancer drugs such as cisplatin or doxorubicin could increment their anticancer effect, enhance their cytotoxic activity toward multi-drug resistant cells, and reduce their toxicity.

Thonningianin A from *Thonningia sanguinea* inhibited the proliferation of HepG-2 human hepatocellular carcinoma cells [123]. Thonningianin A induced caspase-dependent apoptotic cell death, accompanied by an increase in the sub-G1 cell population and DNA fragmentation. Several mechanisms contributing to the antitumor effects were identified: thonningianin A disrupted the mitochondrial membrane potential promoting an increased generation of reactive oxygen species, downregulated the Bcl-xL mRNA expression, induced cell-cycle arrest by changing the cyclin D1 and CDK4 mRNA expression levels. Furthermore, thonningianin A significantly downregulated the NF-κB cell survival pathway concomitantly with the upregulation of the expression level of phosphorylated P38 and downregulation of the expression level of phosphorylated ERK.

### 5.6. Cervical Cancer

Cervical cancer has long remained a leading cause of malignancies-related death in women from United States of America. However, the number of cervical cancer patients and associated death toll has significantly decreased since last few decades, probably due to the regular Human Papilloma Virus (HPV) screening [124]. Apart from other risk factors, strong association exists between cervical cancers and HPV infection, and HPVs are indicated as central etiological factor in incidents of cervical cancer, globally [125]. Ramasamy *et al.*, [126] found that *Phyllanthus watsonii* extract induced apoptosis in HPV-transformed CaSki epidermoid cervical carcinoma cells, and attributed to the high ellagic content its cytotoxic effect. Raspberry extracts naturally enriched with ET inhibit proliferation of cervical cancer cells (HeLa) in a dose-dependent manner [127]. The study further reported the bound ET-enriched fraction of raspberry extracts as more effective (IC50 = 13 µg/mL) than the unbound anthocyanin-enriched fraction (IC50 = 67 µg/mL).

Hydrolysable tannins improve dysfunctional gap junctions communication, which are involved in carcinogenesis. Tellimagrandin I and chebulinic acid restore dysfunctional gap junctions in HeLa cells. *In vitro* exposure of HeLa cells to tellimagrandin I inhibits their proliferation as well as their substrate-independent growth [128].

Camelliin B, the hydrolysable tannin isolated from a non-edible plant (*i.e.*, *Gordonia axillaris* or fried eggplant), is another example of phytochemical useful for cervical cancer treatment. Camelliin B isolated from *G. axillaris* inhibited the growth of HeLa cells with an IC50 of 46.3 µg/mL as compared to the IC50 of 108.0 µg/mL observed in normal cervical fibroblasts [129]. The study showed that camelliin B induces chromatin condensation, a hallmark of apoptosis. Furthermore, camelliin B also exhibited DNA fragmentation properties and inhibited the DNA repair-associated enzyme poly (ADP-ribose) polymerase in HeLa cells. Walnut extracts rich in tellimagrandin I and II induce cytotoxic effects in human HeLa cancer cells by reducing mitochondrial respiration and promoting apoptosis [130]. Ellagic acid was shown to induce G1 arrest via induction of p21 and apoptosis in CaSki human cervix carcinoma cells [59].

The elevated risk of cervical cancer in cigarette smokers is thought to depend on the increased mutations in cervical cells caused by the persistence of smoke habit-associated DNA damage in the presence of HPV infection. Importantly, ellagic acid significantly attenuates cigarette smoke-induced DNA damage in HPV16-transformed human ECT1/E6 E7 ectocervical cells [131], an effect which is likely to derive from ellagic acid antioxidant and free-radical scavenging activity and that further support its chemopreventive potential.

### 5.7. Lung Cancer

Lung cancer is the most prevalent cancer worldwide [68]. The prognosis of lung cancer patients is still poor, and while it is not the most frequently diagnosed cancer in the United States, it is by far the leading cause of cancer-related deaths in the US and also worldwide. Therefore, advances in the treatment of lung cancer are urgently needed.

Although the relative importance of its major constituents, ET and anthocyanins, was not addressed, pomegranate extracts have been found to exert antiproliferative and chemopreventive activities against lung cancer *in vitro* and in animal models [132,133]. Other reports suggest a specific and important role for ET in the pomegranate extract activity against this type of malignancy, in both *in vitro* and animal experimental settings. In a study focusing on purified ellagic acid and punicalagin, Zahin *et al.*, [134] demonstrated that these two compounds were antimutagenic, prevented the formation of benzo[a]pyrene-induced DNA adducts, and were antiproliferative in non-small cell lung cancer A549 and H1299 lung cancer cells. It is worth noting that punicalagin, using the same toxicity tetrazolium assay, had been shown to be far less antiproliferative toward the same A549 cell line [135] as compared to the data reported by Zahin *et al*, [134]. This apparent discrepancy, which points to the importance of standardizing the experimental settings in this kind of studies, is likely to depend on the post-treatment incubation times before determining cell viability: in the first study, cell viability was determined at 24 h [121], while in the second one at 48 h [122], a time which allows a more accurate estimate of the growth inhibitory activity. Kuo *et al.*, [136] found that the ET casuarinin from the bark of *Terminalia arjuna* induced apoptosis in human breast adenocarcinoma MCF-7 cells and in A549 cells by blocking cell-cycle progression in the G0/G1 phase.

Similarly, *jamun* (*Syzygium cumini* L.) seeds and pulp hydrolyzed extracts have been reported to exert antiproliferative activity in A549 cells, which has been associated with the presence of ellagic acid [93].

### 5.8. Skin Cancer

Prolonged exposure of skin to UV radiation is causally linked to several pathological conditions, including photo-aging and photocarcinogenesis. UV damage is partly attributable to increased skin reactive oxygen species generation. Pomegranate fruit extract, which contains very high amounts of ET, has been shown to exert a significant protective effect against UV rays insult and pathological consequences. Orally-administered pomegranate extract containing 90% ellagic acid, by virtue of its antioxidant activity, has been shown to inhibit skin pigmentation induced by exposure to UV radiation in brown guinea pigs [137]; under the same conditions, the extract decreased melanocyte proliferation and melanin synthesis via inhibition of tyrosinase activity to a degree comparable to that of arbutin, an established tyrosinase inhibitor.

Several studies have confirmed the ability of standardized pomegranate extract and pomegranate ET (500–10,000 mg/L) to inhibit free radical generation in UVA- and UVB-irradiated human skin, thus protecting it from DNA fragmentation, skin burns, and pigmentation, and finally decreasing the risk of malignant transformation [4]. Various mechanisms involved include reduction of DNA damage, prevention of UVB-caused matrix metalloproteinases induction, inhibition of matrix metalloproteinases 2 and 9 activity, and decrease in UVB-induced c-Fos protein expression and c-Jun phosphorylation [138].

Animal studies further confirmed the chemopreventive and anticancer activity of ET-rich pomegranate extract: in a UVB initiation-promotion protocol, SKH-1 hairless mice receiving oral pomegranate extract supplementation showed reduced tumor incidence, prolonged latency periods of tumor appearance, and lower tumor body burden compared to that of unsupplemented UVB-irradiated control animals [139].

## 6. Risks and Safe Consumption Levels

In contrast with the widely accepted notion that ET, similarly to other phytochemicals, are health-promoting, chemopreventive, and therapeutically-valuable compounds, data emerged from some studies raised the question of the safety of their consumption [140]. In general, tannins may be toxic to cells and tissues because of their protein precipitation, enzymes inhibition, and mineral binding properties [140,141]. Furthermore, it was reported that pomegranate hydroalcoholic extract exerts mutagenic, genotoxic and clastogenic effects in a panel of *in vitro* and *in vivo* assays [142]. In Chinese hamster B14 cells, ellagic acid and gallic acid caused the production of DNA single-strand breaks with no relation to the concentration used, cytotoxic effects and increased lipid bilayer fluidity, an event which the authors suggested as contributing to DNA single-strand breakage [143]. However, these results are controversial and contradicted by studies demonstrating the lack of mutagenicity of ellagic acid in similar experimental settings [144] and by the hundreds of reports on the DNA protective activity of polyphenols, including ET, against established genotoxic agents.

A study conducted by Filippich *et al.*, [145] linked the generation of lesions on mice liver, early and severe liver necrosis, to punicalagin. However, an update on punicalagin risk assessment revealed neither hepatotoxic nor nephrotoxic effects following sub-chronic oral exposure (6% daily) to Sprague–Dawley rats [146].

ET have been reported to act as α-glucosidase inhibitors and, thus, proposed as adjunctive agents in type-2 diabetes management [147]: a caveat has been associated with this property since the dietary intake of any α-glucosidase inhibitor in normal circumstances might generate risks of carbohydrate malabsorption, gastrointestinal discomfort, flatulence, and diarrhea, such as for acarbose [148,149]. However, to the best of our knowledge, there is no report of such side effects causally linked to ingestion of ET-rich food and fruits.

ET, alongside the condensed tannins, could be considered as antinutritional in animal diets due to their ability of interacting with protein and inhibiting certain enzymes. Antinutritional effects have been reported in animal models, where diet carrying tannins at dosages higher than 10 g/kg b.w. affected animal growth and digestive capacity [150]. However, levels ≥10 g/kg b.w. are unlikely to be attained using standard nutritional regimens; furthermore, a study conducted for risk assessment of chestnut hydrolysable tannins included in lamb diet revealed the lack of any toxic response in terms of weight gain, protein conversion efficiency, and histopathological features [151].

To date, incomplete information is available on toxicity and risk assessment of individual ET. However, the no observed effect levels (NOEL) and no observed adverse effect levels (NOAEL) as determined in some reports are unlikely to portray dietary consumption-associated toxicity. For example, a 90-day sub-chronic toxicity study performed in F344 rats showed that ellagic acid NOEL was 3011 mg/kg b.w./day for males and the NOAEL and NOEL in females were 3254 mg/kg b.w./day and ≤778 mg/kg b.w./day, respectively, and there were no obvious histopathological changes in any of the groups [152]. A 90-day sub-chronic study showed that the LD50 of a standardized pomegranate fruit extract containing 30% punicalagin in Wistar rats was >5 g/kg b.w., with no visible sign of toxicity in terms of feed consumption, weight gain, ophthalmic, and pathological evaluation [153].

Dietary intake of ET varies among cultures, communities and region as has been evidently documented in studies from different countries [6,154]. A global report on the dietary consumption of phytonutrients reveals that peoples from Western Europe have maximum ellagic acid consumption trends in both genders (7.6 mg/day in males and 7.9 mg/day in females). Berries account approximately for 90% of the daily ellagic acid intake [154]. A few reports on the nutritional habits of German and Finnish communities indicate that consumption of berries provides up to 5 mg and 12 mg ET per day, respectively [6,155,156]. Correlating ET consumption trends from various dietary sources with the so far identified NOEL or NOAEL for these biomolecules undoubtedly indicate that ET pose negligible threats to the safety and health security of the consumers, consolidating the notion that ET, either in individual or composite form, can potentially be exploited as health-promoting and potential chemopreventive phytonutrients.

As a final consideration, it could be speculated that an increasing use of ET as anticancer agents could pave the way to the adoption of administration routes different from oral one, such as the intravenous administration: such a route, however, would need to be characterized from the toxicological point of view since this kind of data is still lacking.

## 7. Concluding Remarks

The increasing awareness and knowledge of the capacity of plant-derived compounds to modify cell transformation and cancer cell growth suggest that they could serve as new tools for either preventive and therapeutic interventions. Today, ET are recognized as a class of phytochemicals characterized by a strong potential for development as chemopreventive, and possibly as therapeutic, agents against various human cancers. This could have a direct clinical and translational relevance to cancer patients if consumption of ET-rich fruits and vegetables will unequivocally prove to contrast the process of carcinogenesis and tumor growth, with positive outcomes in terms of survival and quality of life of the patient. To this end, future research should be addressed to define the actual clinical potential of ET through specific studies such as the determination of the systemic bioavailability from either food sources or concentrated formulations, the optimal period of administration and dosing, the toxicity and side effects (if any), the anticancer activity. The effects of single ET and of rational combinations of different ET should also be addressed. A multidisciplinary and coordinated approach will be needed and will involve basic research investigations, epidemiological and preclinical studies including the effect of combining ET with conventional antineoplastic drugs.

## References

[1] Siegel R., Ward E., Brawley O., Jemal A. (2011). The impact of eliminating socioeconomic and racial disparities on premature cancer deaths. CA Cancer J. Clin..

[2] Durko L., Malecka-Panas E. (2014). Lifestyle modifications and colorectal cancer. Curr. Colorectal. Cancer Rep..

[3] Quideau S., Deffieux D., Douat-Casassus C., Pouysegu L. (2011). Plant polyphenols: Chemical properties, biological activities, and synthesis. Angew. Chem. Int. Ed. Engl..

[4] Ismail T., Sestili P., Akhtar S. (2012). Pomegranate peel and fruit extracts: A review of potential anti-inflammatory and anti-infective effects. J. Ethnopharmacol..

[5] Landete J. (2011). Ellagitannins, ellagic acid and their derived metabolites: A review about source, metabolism, functions and health. Food Res. Int..

[6] Koponen J.M., Happonen A.M., Mattila P.H., Torronen A.R. (2007). Contents of anthocyanins and ellagitannins in selected foods consumed in Finland. J. Agric. Food Chem..

[7] Mullen W., McGinn J., Lean M.E., MacLean M.R., Gardner P., Duthie G.G., Yokota T., Crozier A. (2002). Ellagitannins, flavonoids, and other phenolics in red raspberries and their contribution to antioxidant capacity and vasorelaxation properties. J. Agric. Food Chem..

[8] Mullen W., Stewart A.J., Lean M.E., Gardner P., Duthie G.G., Crozier A. (2002). Effect of freezing and storage on the phenolics, ellagitannins, flavonoids, and antioxidant capacity of red raspberries. J. Agric. Food Chem..

[9] Gancel A.L., Feneuil A., Acosta O., Perez A.M., Vaillant F. (2011). Impact of industrial processing and storage on major polyphenols and the antioxidant capacity of tropical highland blackberry (*Rubus adenotrichus*). Food Res. Int..

[10] Hager T.J., Howard L.R., Liyanage R., Lay J.O., Prior R.L. (2008). Ellagitannin composition of blackberry as determined by HPLC-ESI-MS and MALDI-TOF-MS. J. Agric. Food Chem..

[11] Mertz C., Cheynier V., Gunata Z., Brat P. (2007). Analysis of phenolic compounds in two blackberry species (*Rubus glaucus* and *Rubus adenotrichus*) by high-performance liquid chromatography with diode array detection and electrospray ion trap mass spectrometry. J. Agric. Food Chem..

[12] Lansky E.P., Newman R.A. (2007). *Punica granatum* (pomegranate) and its potential for prevention and treatment of inflammation and cancer. J. Ethnopharmacol..

[13] Beekwilder J., Jonker H., Meesters P., Hall R.D., van der Meer I.M., de Vos C.H.R. (2005). Antioxidants in raspberry: On-line analysis links antioxidant activity to a diversity of individual metabolites. J. Agric. Food Chem..

[14] Lee J.H., Johnson J.V., Talcott S.T. (2005). Identification of ellagic acid conjugates and other polyphenolics in muscadine grapes by HPLC-ESI-MS. J. Agric. Food Chem..

[15] Maatta-Riihinen K.R., Kamal-Eldin A., Torronen A.R. (2004). Identification and quantification of phenolic compounds in berries of *Fragaria* and *Rubus* species (family Rosaceae). J. Agric. Food Chem..

[16] Mullen W., Yokota T., Lean M.E., Crozier A. (2003). Analysis of ellagitannins and conjugates of ellagic acid and quercetin in raspberry fruits by LC-MS^n^. Phytochemistry.

[17] Rangkadilok N., Worasuttayangkurn L., Bennett R.N., Satayavivad J. (2005). Identification and quantification of polyphenolic compounds in longan (*Euphoria longana* Lam.) fruit. J. Agric. Food Chem..

[18] Seeram N.P., Lee R., Heber D. (2004). Bioavailability of ellagic acid in human plasma after consumption of ellagitannins from pomegranate (*Punica granatum* L.) juice. Clin. Chim. Acta.

[19] Abe L.T., Lajolo F.M., Genovese M.I. (2010). Comparison of phenol content and antioxidant capacity of nuts. Food Sci. Technol. (Camp.).

[20] Kaume L., Howard L.R., Devareddy L. (2012). The blackberry fruit: A review on its composition and chemistry, metabolism and bioavailability, and health benefits. J. Agric. Food Chem..

[21] Shi N., Clinton S.K., Liu Z., Wang Y., Riedl K.M., Schwartz S.J., Zhang X., Pan Z., Chen T. (2015). Strawberry phytochemicals inhibit azoxymethane/dextran sodium sulfate-induced colorectal carcinogenesis in Crj: CD-1 mice. Nutrients.

[22] Yoshida T., Ito H., Hatano T., Kurata M., Nakanishi T., Inada A., Murata H., Inatomi Y., Matsuura N., Ono K. (1996). New hydrolyzable tannins, Shephagenins A and B, from shepherdia argentea as HIV-1 reverse transcriptase inhibitors. Chem. Pharm. Bull..

[23] Yoshida T., Hatano T., Ito H. (2000). Chemistry and function of vegetable polyphenols with high molecular weights. BioFactors.

[24] Harborne J.B. (1983). Plant Phenolics.

[25] Haslam E. (1989). Plant Polyphenols: Vegetable Tannins Revisited.

[26] Haslam E., Cai Y. (1994). Plant polyphenols (vegetable tannins): Gallic acid metabolism. Nat. Prod. Rep..

[27] Khanbabaee K., van Ree T. (2001). Tannins: Classification and definition. Nat. Prod. Rep..

[28] Salminen J.P., Karonen M. (2011). Chemical ecology of tannins and other phenolics: We need a change in approach. Funct. Ecol..

[29] Gross G.G. (2008). From lignins to tannins: Forty years of enzyme studies on the biosynthesis of phenolic compounds. Phytochemistry.

[30] Larrosa M., García-Conesa M.T., Espín J.C., Tomás-Barberán F.A. (2012). Bioavailability and Metabolism of Ellagic Acid and Ellagitannins.

[31] Larrosa M., Tomas-Barberan F.A., Espin J.C. (2006). The dietary hydrolysable tannin punicalagin releases ellagic acid that induces apoptosis in human colon adenocarcinoma Caco-2 cells by using the mitochondrial pathway. J. Nutr. Biochem..

[32] Kilkowski W.J., Gross G.G. (1999). Color reaction of hydrolyzable tannins with bradford reagent, coomassie brilliant blue. Phytochemistry.

[33] Barbehenn R.V., Jones C.P., Hagerman A.E., Karonen M., Salminen J.P. (2006). Ellagitannins have greater oxidative activities than condensed tannins and galloyl glucoses at high pH: Potential impact on caterpillars. J. Chem. Ecol..

[34] Barbehenn R.V., Jones C.P., Karonen M., Salminen J.P. (2006). Tannin composition affects the oxidative activities of tree leaves. J. Chem. Ecol..

[35] Quideau S. (2009). Chemistry and Biology of Ellagitannins: An Underestimated Class of Bioactive Plant Polyphenols.

[36] Okuda T., Yoshida T., Hatano T. (1995). Hydrolyzable tannins and related polyphenols. Fortschritte der Chemie Organischer Naturstoffe/Progress in the Chemistry of Organic Natural Products.

[37] Okuda T., Yoshida T., Hatano T. (2000). Correlation of oxidative transformations of hydrolyzable tannins and plant evolution. Phytochemistry.

[38] Okuda T., Yoshida T., Ashida M., Yazaki K. (1983). Tannis of *Casuarina* and *Stachyurus* species. Part 1. Structures of pendunculagin, casuarictin, strictinin, casuarinin, casuariin, and stachyurin. J. Chem. Soc. Perkin Trans..

[39] Okuda T., Yoshida T., Kuwahara M., Memon M.U., Shingu T. (1982). Agrimoniin and potentillin, an ellagitannin dimer and monomer having an α-glucose core. J. Chem. Soc. Chem. Commun..

[40] Okuda T., Yoshida T., Kuwahara M., Memon M.U., Shingu T. (1984). Tannins of rosaceous medicinal plants. I. Structures of potentillin, agrimonic acids A and B, and agrimoniin, a dimeric ellagitannin. Chem. Pharm. Bull..

[41] Lipińska L., Klewicka E., Sójka M. (2014). Structure, occurrence and biological activity of ellagitannins: A general review. Acta Sci. Pol. Technol. Aliment..

[42] Kaneshima T., Myoda T., Nakata M., Fujimori T., Toeda K., Nishizawa M. (2016). Antioxidant activity of *C*-glycosidic ellagitannins from the seeds and peel of camu-camu (*Myrciaria dubia*). LWT Food Sci. Technol..

[43] Tanaka T., Ueda N., Shinohara H., Nonaka G.-I., Kouno I. (1997). Four new *C*-glycosidic ellagitannins, castacrenins DG, from Japanese chestnut wood (castanea crenata SIEB. Et ZUCC.). Chem. Pharm. Bull..

[44] Omar M., Matsuo Y., Maeda H., Saito Y., Tanaka T. (2014). New metabolites of *C*-glycosidic ellagitannin from Japanese oak sapwood. Org Lett..

[45] Jiang Z.-H., Tanaka T., Kouno I. (1999). Three novel *C*-glycosidic ellagitannins, Rhoipteleanins H, I, and J, from Rhoiptelea c hiliantha. J. Nat. Prod..

[46] Quideau S., Jourdes M., Lefeuvre D., Montaudon D., Saucier C., Glories Y., Pardon P., Pourquier P. (2005). The chemistry of wine polyphenolic *C*-glycosidic ellagitannins targeting human topoisomerase II. Chemistry.

[47] Clifford M.N., Scalbert A. (2000). Ellagitannins—Nature, occurrence and dietary burden. J. Sci. Food Agric..

[48] Garcia-Munoz C., Vaillant F. (2014). Metabolic fate of ellagitannins: Implications for health, and research perspectives for innovative functional foods. Crit. Rev. Food Sci. Nutr..

[49] Bialonska D., Kasimsetty S.G., Khan S.I., Ferreira D. (2009). Urolithins, intestinal microbial metabolites of pomegranate ellagitannins, exhibit potent antioxidant activity in a cell-based assay. J. Agric. Food Chem..

[50] González-Barrio R.O., Borges G., Mullen W., Crozier A. (2010). Bioavailability of anthocyanins and ellagitannins following consumption of raspberries by healthy humans and subjects with an ileostomy. J. Agric. Food Chem..

[51] Garcia-Munoz C., Hernàndez L., Pèrez A., Vaillant F. (2014). Diversity of urinary excretion patterns of main ellagitannins’ colonic metabolites after ingestion of tropical highland blackberry (*Rubus adenotrichus*) juice. Food Res. Int..

[52] Seeram N.P., Lee R., Scheuller H.S., Heber D. (2006). Identification of phenolic compounds in strawberries by liquid chromatography electrospray ionization mass spectroscopy. Food Chem..

[53] Cerdá B., Espín J.C., Parra S., Martínez P., Tomás-Barberán F.A. (2004). The potent *in vitro* antioxidant ellagitannins from pomegranate juice are metabolised into bioavailable but poor antioxidant hydroxy-6*H*-dibenzopyran-6-one derivatives by the colonic microflora of healthy humans. Eur. J. Nutr..

[54] Larrosa M., Garcia-Conesa M.T., Espin J.C., Tomas-Barberan F.A. (2010). Ellagitannins, ellagic acid and vascular health. Mol. Asp. Med..

[55] Heber D. (2008). Multitargeted therapy of cancer by ellagitannins. Cancer Lett..

[56] Tomás-Barberán F.A., García-Villalba R., González-Sarrías A., Selma M.V., Espín J.C. (2014). Ellagic acid metabolism by human gut microbiota: Consistent observation of three urolithin phenotypes in intervention trials, independent of food source, age, and health status. J. Agric. Food Chem..

[57] Nicoli M., Anese M., Parpinel M. (1999). Influence of processing on the antioxidant properties of fruit and vegetables. Trends Food Sci. Technol..

[58] Balkwill F., Coussens L.M. (2004). Cancer: An inflammatory link. Nature.

[59] Narayanan B.A., Geoffroy O., Willingham M.C., Re G.G., Nixon D.W. (1999). p53/p21(WAF1/CIP1) expression and its possible role in G1 arrest and apoptosis in ellagic acid treated cancer cells. Cancer Lett..

[60] Vanella L., di Giacomo C., Acquaviva R., Barbagallo I., Cardile V., Kim D.H., Abraham N.G., Sorrenti V. (2013). Apoptotic markers in a prostate cancer cell line: Effect of ellagic acid. Oncol. Rep..

[61] Vicinanza R., Zhang Y., Henning S.M., Heber D. (2013). Pomegranate juice metabolites, ellagic acid and urolithin a, synergistically inhibit androgen-independent prostate cancer cell growth via distinct effects on cell cycle control and apoptosis. Evid. Based Complement. Altern. Med..

[62] Chen H.-S., Bai M.-H., Zhang T., Li G.-D., Liu M. (2015). Ellagic acid induces cell cycle arrest and apoptosis through TGF-β/Smad3 signaling pathway in human breast cancer MCF-7 cells. Int. J. Oncol..

[63] Wen X.Y., Wu S.Y., Li Z.Q., Liu Z.Q., Zhang J.J., Wang G.F., Jiang Z.H., Wu S.G. (2009). Ellagitannin (BJA3121), an anti-proliferative natural polyphenol compound, can regulate the expression of miRNAs in HepG2 cancer cells. Phytother. Res..

[64] Seeram N.P., Adams L.S., Henning S.M., Niu Y., Zhang Y., Nair M.G., Heber D. (2005). *In vitro* antiproliferative, apoptotic and antioxidant activities of punicalagin, ellagic acid and a total pomegranate tannin extract are enhanced in combination with other polyphenols as found in pomegranate juice. J. Nutr. Biochem..

[65] Sartippour M.R., Seeram N.P., Rao J.Y., Moro A., Harris D.M., Henning S.M., Firouzi A., Rettig M.B., Aronson W.J., Pantuck A.J. (2008). Ellagitannin-rich pomegranate extract inhibits angiogenesis in prostate cancer *in vitro* and *in vivo*. Int. J. Oncol..

[66] Lee S.-J., Lee H.-K. (2005). Sanguiin H-6 blocks endothelial cell growth through inhibition of VEGF binding to VEGF receptor. Arch. Pharmacal. Res..

[67] Gambari R., Hau D.K.P., Wong W.Y., Chui C.H. (2014). Sensitization of Hep3B hepatoma cells to cisplatin and doxorubicin by corilagin. Phytotherapy Res..

[68] CDC 2012 Top Ten Cancers. https://nccd.cdc.gov/uscs/toptencancers.aspx.

[69] Masko E.M., Allott E.H., Freedland S.J. (2013). The relationship between nutrition and prostate cancer: Is more always better?. Eur. Urol..

[70] Cohen J.H., Kristal A.R., Stanford J.L. (2000). Fruit and vegetable intakes and prostate cancer risk. J. Natl. Cancer Inst..

[71] Kolonel L.N., Hankin J.H., Whittemore A.S., Wu A.H., Gallagher R.P., Wilkens L.R., John E.M., Howe G.R., Dreon D.M., West D.W. (2000). Vegetables, fruits, legumes and prostate cancer: A multiethnic case-control study. Cancer Epidemiol. Biomark. Prev..

[72] Seeram N.P., Aronson W.J., Zhang Y., Henning S.M., Moro A., Lee R.-P., Sartippour M., Harris D.M., Rettig M., Suchard M.A. (2007). Pomegranate ellagitannin-derived metabolites inhibit prostate cancer growth and localize to the mouse prostate gland. J. Agric. Food Chem..

[73] Albrecht M., Jiang W., Kumi-Diaka J., Lansky E.P., Gommersall L.M., Patel A., Mansel R.E., Neeman I., Geldof A.A., Campbell M.J. (2004). Pomegranate extracts potently suppress proliferation, xenograft growth, and invasion of human prostate cancer cells. J. Med. Food.

[74] Malik A., Afaq F., Sarfaraz S., Adhami V.M., Syed D.N., Mukhtar H. (2005). Pomegranate fruit juice for chemoprevention and chemotherapy of prostatesystemic antioxidant propo cancer. Proc. Natl. Acad. Sci. USA.

[75] Stolarczyk M., Piwowarski J.P., Granica S., Stefanska J., Naruszewicz M., Kiss A.K. (2013). Extracts from *Epilobium* sp. Herbs, their components and gut microbiota metabolites of epilobium ellagitannins, urolithins, inhibit hormone-dependent prostate cancer cells-(lNCaP) proliferation and PSA secretion. Phytother. Res..

[76] Stolarczyk M., Naruszewicz M., Kiss A.K. (2013). Extracts from *Epilobium* sp. Herbs induce apoptosis in human hormone-dependent prostate cancer cells by activating the mitochondrial pathway. J. Pharm. Pharmacol..

[77] Walia H., Arora S. (2013). Terminalia chebula—A pharmacognistic account. J. Med. Plant Res..

[78] Saleem A., Husheem M., Harkonen P., Pihlaja K. (2002). Inhibition of cancer cell growth by crude extract and the phenolics of *Terminalia chebula* retz. Fruit. J. Ethnopharmacol..

[79] Calixto J.B. (2005). Twenty-five years of research on medicinal plants in Latin America: A personal view. J. Ethnopharmacol..

[80] Eberhart C.E., Coffey R.J., Radhika A., Giardiello F.M., Ferrenbach S., Dubois R.N. (1994). Up-regulation of cyclooxygenase 2 gene expression in human colorectal adenomas and adenocarcinomas. Gastroenterology.

[81] Fajardo A.M., Piazza G.A. (2015). Chemoprevention in gastrointestinal physiology and disease. Anti-inflammatory approaches for colorectal cancer chemoprevention. Am. J. Physiol. Gastrointest. Liver Physiol..

[82] Madka V., Rao C.V. (2013). Anti-inflammatory phytochemicals for chemoprevention of colon cancer. Curr. Cancer Drug Targets.

[83] Adams L.S., Seeram N.P., Aggarwal B.B., Takada Y., Sand D., Heber D. (2006). Pomegranate juice, total pomegranate ellagitannins, and punicalagin suppress inflammatory cell signaling in colon cancer cells. J. Agric. Food Chem..

[84] Kasimsetty S.G., Bialonska D., Reddy M.K., Ma G., Khan S.I., Ferreira D. (2010). Colon cancer chemopreventive activities of pomegranate ellagitannins and urolithins. J. Agric. Food Chem..

[85] Sharma M., Li L., Celver J., Killian C., Kovoor A., Seeram N.P. (2009). Effects of fruit ellagitannin extracts, ellagic acid, and their colonic metabolite, urolithin a, on Wnt signaling. J. Agric. Food Chem..

[86] CDC Breast Cancer Statistics. http://www.cdc.gov/cancer/breast/statistics/.

[87] Russo I.H., Russo J. (1998). Role of hormones in mammary cancer initiation and progression. J. Mammary Gland Biol. Neoplasia.

[88] Gebre-Medhin M., Kindblom L.-G., Wennbo H., Törnell J., Meis-Kindblom J.M. (2001). Growth hormone receptor is expressed in human breast cancer. Am. J. Pathol..

[89] Chen Z., Gu K., Zheng Y., Zheng W., Lu W., Shu X.O. (2008). The use of complementary and alternative medicine among Chinese women with breast cancer. J. Altern. Complement. Med..

[90] Brodie A., Sabnis G., Jelovac D. (2006). Aromatase and breast cancer. J. Steroid Biochem. Mol. Biol..

[91] Chen S. (1998). Aromatase and breast cancer. Front. Biosci..

[92] Kim N.D., Mehta R., Yu W., Neeman I., Livney T., Amichay A., Poirier D., Nicholls P., Kirby A., Jiang W. (2002). Chemopreventive and adjuvant therapeutic potential of pomegranate (*Punica granatum*) for human breast cancer. Breast Cancer Res. Treat..

[93] Aqil F., Gupta A., Munagala R., Jeyabalan J., Kausar H., Sharma R.J., Singh I.P., Gupta R.C. (2012). Antioxidant and antiproliferative activities of anthocyanin/ellagitannin-enriched extracts from *Syzygium cumini* L. (Jamun, the Indian Blackberry). Nutr. Cancer.

[94] Li L., Adams L.S., Chen S., Killian C., Ahmed A., Seeram N.P. (2009). *Eugenia jambolana* lam. Berry extract inhibits growth and induces apoptosis of human breast cancer but not non-tumorigenic breast cells. J. Agric. Food Chem..

[95] Shi L., Gao X., Li X., Jiang N., Luo F., Gu C., Chen M., Cheng H., Liu P. (2015). Ellagic acid enhances the efficacy of PI3K inhibitor GDC-0941 in breast cancer cells. Curr. Mol. Med..

[96] Barrajón-Catalán E., Fernández-Arroyo S., Saura D., Guillén E., Fernández-Gutiérrez A., Segura-Carretero A., Micol V. (2010). Cistaceae aqueous extracts containing ellagitannins show antioxidant and antimicrobial capacity, and cytotoxic activity against human cancer cells. Food Chem. Toxicol..

[97] Miyamoto K.I., Nomura M., Sasakura M., Matsui E., Koshiura R., Murayama T., Furukawa T., Hatano T., Yoshida T., Okuda T. (1993). Antitumor activity of oenothein B, a unique macrocyclic ellagitannin. Jpn. J. Cancer Res. Gann.

[98] Enzinger P.C., Mayer R.J. (2003). Esophageal cancer. N. Engl. J. Med..

[99] De Stefani E., Barrios E., Fierro L. (1993). Black (air-cured) and blond (flue-cured) tobacco and cancer risk. III: Oesophageal cancer. Eur. J. Cancer.

[100] Stoner G.D., Chen T., Kresty L.A., Aziz R.M., Reinemann T., Nines R. (2006). Protection against esophageal cancer in rodents with lyophilized berries: Potential mechanisms. Nutr. Cancer.

[101] Kresty L.A., Morse M.A., Morgan C., Carlton P.S., Lu J., Gupta A., Blackwood M., Stoner G.D. (2001). Chemoprevention of esophageal tumorigenesis by dietary administration of lyophilized black raspberries. Cancer Res..

[102] Bishayee A., Haskell Y., Do C., Siveen K.S., Mohandas N., Sethi G., Stoner G.D. (2015). Potential benefits of edible berries in the management of aerodigestive and gastrointestinal tract cancers: Preclinical and clinical evidence. Crit. Rev. Food Sci. Nutr..

[103] Mandal S., Stoner G.D. (1990). Inhibition of *N*-nitrosobenzylmethylamine-induced esophageal tumorigenesis in rats by ellagic acid. Carcinogenesis.

[104] Daniel E.M., Stoner G.D. (1991). The effects of ellagic acid and 13-cis-retinoic acid on *N*-nitrosobenzylmethylamine-induced esophageal tumorigenesis in rats. Cancer Lett..

[105] Stoner G.D., Morse M.A. (1997). Isothiocyanates and plant polyphenols as inhibitors of lung and esophageal cancer. Cancer Lett..

[106] Wang L.S., Dombkowski A.A., Seguin C., Rocha C., Cukovic D., Mukundan A., Henry C., Stoner G.D. (2011). Mechanistic basis for the chemopreventive effects of black raspberries at a late stage of rat esophageal carcinogenesis. Mol. Carcinog..

[107] Wang L.S., Hecht S., Carmella S., Seguin C., Rocha C., Yu N., Stoner K., Chiu S., Stoner G. (2010). Berry ellagitannins may not be sufficient for prevention of tumors in the rodent esophagus. J. Agric. Food Chem..

[108] Liu H., Li J., Zhao W., Bao L., Song X., Xia Y., Wang X., Zhang C., Wang X., Yao X. (2009). Fatty acid synthase inhibitors from *Geum japonicum* Thunb. var. chinense. Chem. Biodivers..

[109] Zhang Y., Seeram N.P., Lee R., Feng L., Heber D. (2008). Isolation and identification of strawberry phenolics with antioxidant and human cancer cell antiproliferative properties. J. Agric. Food Chem..

[110] Weisburg J.H., Schuck A.G., Reiss S.E., Wolf B.J., Fertel S.R., Zuckerbraun H.L., Babich H. (2013). Ellagic acid, a dietary polyphenol, selectively cytotoxic to HSC-2 oral carcinoma cells. Anticancer Res..

[111] Zhu X., Xiong L., Zhang X., Shi N., Zhang Y., Ke J., Sun Z., Chen T. (2015). Lyophilized strawberries prevent 7, 12-dimethylbenz [α] anthracene (DMBA)-induced oral squamous cell carcinogenesis in hamsters. J. Funct. Foods.

[112] Casto B.C., Knobloch T.J., Galioto R.L., Yu Z., Accurso B.T., Warner B.M. (2013). Chemoprevention of oral cancer by lyophilized strawberries. Anticancer Res..

[113] Priyadarsini R.V., Kumar N., Khan I., Thiyagarajan P., Kondaiah P., Nagini S. (2012). Gene expression signature of DMBA-induced hamster buccal pouch carcinomas: Modulation by chlorophyllin and ellagic acid. PLoS ONE.

[114] Anitha P., Priyadarsini R.V., Kavitha K., Thiyagarajan P., Nagini S. (2013). Ellagic acid coordinately attenuates Wnt/β-catenin and NF-κb signaling pathways to induce intrinsic apoptosis in an animal model of oral oncogenesis. Eur. J. Nutr..

[115] Kowshik J., Giri H., Kranthi Kiran Kishore T., Kesavan R., Naik Vankudavath R., Bhanuprakash Reddy G., Dixit M., Nagini S. (2014). Ellagic acid inhibits VEGF/VEGFR2, PI3K/Akt and MAPK signaling cascades in the hamster cheek pouch carcinogenesis model. Anti-Cancer Agents Med. Chem..

[116] Ding Y., Yao H., Yao Y., Fai L.Y., Zhang Z. (2013). Protection of dietary polyphenols against oral cancer. Nutrients.

[117] Naghavi M., Wang H., Lozano R., Davis A., Liang X., Zhou M., Vollset S.E., Ozgoren A.A., Abdalla S., Abd-Allah F. (2015). Global, regional, and national age-sex specific all-cause and cause-specific mortality for 240 causes of death, 1990–2013: A systematic analysis for the global burden of disease study 2013. Lancet.

[118] Oh G.-S., Pae H.-O., Oh H., Hong S.-G., Kim I.-K., Chai K.-Y., Yun Y.-G., Kwon T.-O., Chung H.-T. (2001). *In vitro* anti-proliferative effect of 1,2,3,4,6-penta-*O*-galloyl-beta-d-glucose on human hepatocellular carcinoma cell line, SK-HEP-1 cells. Cancer Lett..

[119] Yin S., Dong Y., Li J., Lü J., Hu H. (2011). Penta-1,2,3,4,6-*O*-galloyl-beta-d-glucose induces senescence-like terminal S-phase arrest in human hepatoma and breast cancer cells. Mol. Carcinog..

[120] Dong Y., Yin S., Jiang C., Luo X., Guo X., Zhao C., Fan L., Meng Y., Lu J., Song X. (2014). Involvement of autophagy induction in penta-1,2,3,4,6-*O*-galloyl-β-d-glucose-induced senescence-like growth arrest in human cancer cells. Autophagy.

[121] Hau D.K.-P., Zhu G.-Y., Leung A.K.-M., Wong R.S.-M., Cheng G.Y.-M., Lai P.B., Tong S.-W., Lau F.-Y., Chan K.-W., Wong W.-Y. (2010). *In vivo* anti-tumour activity of corilagin on Hep3B hepatocellular carcinoma. Phytomedicine.

[122] Ming Y., Zheng Z., Chen L., Zheng G., Liu S., Yu Y., Tong Q. (2013). Corilagin inhibits hepatocellular carcinoma cell proliferation by inducing G2/M phase arrest. Cell Biol. Int..

[123] Zhang T.-T., Yang L., Jiang J.-G. (2015). Effects of thonningianin A in natural foods on apoptosis and cell cycle arrest of HepG-2 human hepatocellular carcinoma cells. Food Funct..

[124] CDC Cervical Cancer Statistics. http://www.cdc.gov/cancer/cervical/statistics/.

[125] Bosch F.X., Manos M.M., Munoz N., Sherman M., Jansen A.M., Peto J., Schiffman M.H., Moreno V., Kurman R., Shah K.V. (1995). Prevalence of human papillomavirus in cervical cancer: A worldwide perspective. J. Natl. Cancer Inst..

[126] Ramasamy S., Abdul Wahab N., Zainal Abidin N., Manickam S., Zakaria Z. (2012). Growth inhibition of human gynecologic and colon cancer cells by phyllanthus watsonii through apoptosis induction. PLoS ONE.

[127] Ross H.A., McDougall G.J., Stewart D. (2007). Antiproliferative activity is predominantly associated with ellagitannins in raspberry extracts. Phytochemistry.

[128] Yi Z.C., Liu Y.Z., Li H.X., Yin Y., Zhuang F.Y., Fan Y.B., Wang Z. (2006). Tellimagrandin I enhances gap junctional communication and attenuates the tumor phenotype of human cervical carcinoma HeLa cells *in vitro*. Cancer Lett..

[129] Wang C.C., Chen L.G., Yang L.L. (2001). Camelliin B induced apoptosis in HeLa cell line. Toxicology.

[130] Le V., Esposito D., Grace M.H., Ha D., Pham A., Bortolazzo A., Bevens Z., Kim J., Okuda R., Komarnytsky S. (2014). Cytotoxic effects of ellagitannins isolated from walnuts in human cancer cells. Nutr. Cancer.

[131] Moktar A., Ravoori S., Vadhanam M.V., Gairola C.G., Gupta R.C. (2009). Cigarette smoke-induced DNA damage and repair detected by the comet assay in HPV-transformed cervical cells. Int. J. Oncol..

[132] Khan N., Afaq F., Kweon M.-H., Kim K., Mukhtar H. (2007). Oral consumption of pomegranate fruit extract inhibits growth and progression of primary lung tumors in mice. Cancer Res..

[133] Khan N., Hadi N., Afaq F., Syed D.N., Kweon M.H., Mukhtar H. (2007). Pomegranate fruit extract inhibits prosurvival pathways in human A549 lung carcinoma cells and tumor growth in athymic nude mice. Carcinogenesis.

[134] Zahin M., Ahmad I., Gupta R.C., Aqil F. (2014). Punicalagin and ellagic acid demonstrate antimutagenic activity and inhibition of benzo [a] pyrene induced DNA adducts. BioMed Res. Int..

[135] Kulkarni A.P., Mahal H., Kapoor S., Aradhya S. (2007). *In vitro* studies on the binding, antioxidant, and cytotoxic actions of punicalagin. J. Agric. Food chem..

[136] Kuo P.-L., Hsu Y.-L., Lin T.-C., Lin L.-T., Chang J.-K., Lin C.-C. (2005). Casuarinin from the bark of *Terminalia arjuna* induces apoptosis and cell cycle arrest in human breast adenocarcinoma MCF-7 cells. Planta Med..

[137] Yoshimura M., Watanabe Y., Kasai K., Yamakoshi J., Koga T. (2005). Inhibitory effect of an ellagic acid-rich pomegranate extract on tyrosinase activity and ultraviolet-induced pigmentation. Biosci. Biotechnol. Biochem..

[138] Afaq F., Zaid M.A., Khan N., Dreher M., Mukhtar H. (2009). Protective effect of pomegranate-derived products on UVB-mediated damage in human reconstituted skin. Exp. Dermatol..

[139] Afaq F., Zaid M., Khan N., Syed D., Yun J.-M., Sarfaraz S., Suh Y., Mukhtar H. Inhibitory effect of oral feeding of pomegranate fruit extract on UVB-induced skin carcinogenesis in SKH-1 hairless mice. Proceedings of the 99th AACR Annual Meeting.

[140] Chung K.-T., Wei C.-I., Johnson M.G. (1998). Are tannins a double-edged sword in biology and health?. Trends Food Sci. Technol..

[141] Mennen L.I., Walker R., Bennetau-Pelissero C., Scalbert A. (2005). Risks and safety of polyphenol consumption. Am. J. Clin. Nutr..

[142] Sánchez-Lamar A., Fonseca G., Fuentes J.L., Cozzi R., Cundari E., Fiore M., Ricordy R., Perticone P., Degrassi F., de Salvia R. (2008). Assessment of the genotoxic risk of *Punica granatum* L.(Punicaceae) whole fruit extracts. J. Ethnopharmacol..

[143] Labieniec M., Gabryelak T. (2003). Effects of tannins on Chinese hamster cell line B14. Mutat. Res. Genet. Toxicol. Environ. Mutagen..

[144] Chen S.C., Chung K.T. (2000). Mutagenicity and antimutagenicity studies of tannic acid and its related compounds. Food Chem. Toxicol..

[145] Filippich L.J., Zhu J., Oelrichs P., Alsalami M.T., Doig A.J., Cao G.R., English P.B. (1991). Hepatotoxic and nephrotoxic principles in terminalia oblongata. Res. Vet. Sci..

[146] Cerdá B., Cerón J.J., Tomás-Barberán F.A., Espín J.C. (2003). Repeated oral administration of high doses of the pomegranate ellagitannin punicalagin to rats for 37 days is not toxic. J. Agric. Food Chem..

[147] McDougall G.J., Shpiro F., Dobson P., Smith P., Blake A., Stewart D. (2005). Different polyphenolic components of soft fruits inhibit α-amylase and α-glucosidase. J. Agric. Food Chem..

[148] Godbout A., Chiasson J.L. (2007). Who should benefit from the use of alpha-glucosidase inhibitors?. Curr. Diabetes Rep..

[149] Li H., Tanaka T., Zhang Y.-J., Yang C.-R., Kouno I. (2007). Rubusuaviins A–F, monomeric and oligomeric ellagitannins from Chinese sweet tea and their α-amylase inhibitory activity. Chem. Pharm. Bull..

[150] Santos-Buelga C., Scalbert A. (2000). Proanthocyanidins and tannin-like compounds—Nature, occurrence, dietary intake and effects on nutrition and health. J. Sci. Food Agric..

[151] Frutos P., Raso M., Hervás G., Mantecón Á.R., Pérez V., Giráldez F.J. (2004). Is there any detrimental effect when a chestnut hydrolysable tannin extract is included in the diet of finishing lambs?. Anim. Res..

[152] Tasaki M., Umemura T., Maeda M., Ishii Y., Okamura T., Inoue T., Kuroiwa Y., Hirose M., Nishikawa A. (2008). Safety assessment of ellagic acid, a food additive, in a subchronic toxicity study using F344 rats. Food Chem. Toxicol..

[153] Patel C., Dadhaniya P., Hingorani L., Soni M. (2008). Safety assessment of pomegranate fruit extract: Acute and subchronic toxicity studies. Food Chem. Toxicol..

[154] Murphy M.M., Barraj L.M., Spungen J.H., Herman D.R., Randolph R.K. (2014). Global assessment of select phytonutrient intakes by level of fruit and vegetable consumption. Br. J. Nutr..

[155] Ovaskainen M.-L., Törrönen R., Koponen J.M., Sinkko H., Hellström J., Reinivuo H., Mattila P. (2008). Dietary intake and major food sources of polyphenols in Finnish adults. J. Nutr..

[156] Radtke J., Linseisen J., Wolfram G. (1998). Phenolic acid intake of adults in a Bavarian subgroup of the national food consumption survey. Z. Ernahrungswiss..

